# Lysophosphatidic Acid Signaling in Late Cleavage and Blastocyst Stage Bovine Embryos

**DOI:** 10.1155/2014/678968

**Published:** 2014-04-15

**Authors:** Ana Catarina Torres, Dorota Boruszewska, Mariana Batista, Ilona Kowalczyk-Zieba, Patricia Diniz, Emilia Sinderewicz, Jean Sebastian Saulnier-Blache, Izabela Woclawek-Potocka, Luis Lopes-da-Costa

**Affiliations:** ^1^Faculty of Veterinary Medicine, CIISA,University of Lisbon, 1300-477 Lisbon, Portugal; ^2^Department of Reproductive Immunology and Pathology, Institute of Animal Reproduction and Food Research, Polish Academy of Sciences, 10-747 Olsztyn, Poland; ^3^Institute of Metabolic and Cardiovascular Diseases, Inserm U1048, Paul Sabatier University, 31432 Toulouse, France

## Abstract

Lysophosphatidic acid (LPA) is a known cell signaling lipid mediator in reproductive tissues. In the cow, LPA is involved in luteal and early pregnancy maintenance. Here, we evaluated the presence and role of LPA in bovine early embryonic development. In relevant aspects, bovine embryos reflect more closely the scenario occurring in human embryos than the mouse model. Transcription of mRNA and protein expression of enzymes involved in LPA synthesis (ATX and *c*PLA_2_) and of LPA receptors (LPAR1–4) were detected in Days 5 and 8 *in vitro* produced embryos. Embryonic LPA production into culture medium was also detected at both stages of development. Supplementation of culture medium with LPA (10^−5^ M) between Days 2 and 8 had no effect on embryo yield and quality and on blastocyst relative mRNA abundance of genes involved in prostaglandin synthesis (*PTGS2*, *PGES*, and *PGFS*) and steroidogenesis (3**β** *HSD*). However, LPA treatment affected transcription levels of embryo quality markers, decreasing *BAX* (apoptotic) and increasing *BCL2* (antiapoptotic) and *IGF2R* (growth marker) gene transcription levels. Blastocyst transcription of *OCT4* (pluripotency marker) was not affected by LPA stimulation. In conclusion, LPA is an early bovine embryonic autocrine/paracrine signaling mediator, and LPA action may be relevant in early embryo-maternal interactions leading to embryonic survival.

## 1. Introduction

Lysophosphatidic acid (LPA) is an extracellular lipid involved in the cellular mediation of a plethora of physiological and pathological events in several tissues of vertebrates. LPA signaling was associated with a broad range of cellular events, including survival, differentiation, proliferation, migration, invasion, and adhesion [1–3].

Two major pathways of LPA production were proposed: the intracellular LPA generation from phosphatidic acid by phospholipase A_1_ or PLA_2_ and the extracellular LPA generation, from lysophosphatidylcholine by autotaxin (ATX), which converts lysophosphatidylcholine to LPA [4, 5]. The diversity of LPA effects on cells is explained by the activation of different signaling pathways associated with different G-protein coupled receptors (LPARs) and their interaction with several types of G proteins (Gq, Gi, Gs, and G12/13) [6]. Initially, three subtypes of endothelial differentiation gene (Edg) family G protein-coupled receptors (LPAR1, LPAR2, and LPAR3) were described. Until the present date, several other LPARs have been identified, structurally distinct from Edg family G receptors, including LPAR4 [7].

Mice null for one or more LPA receptors and for ATX were generated, in order to evaluate the role of LPA signaling [8]. Mutant null mice presented disturbances in uterine embryo spacing and implantation and in spermatogenesis. An increasing number of roles for LPA signaling in reproductive function have been described in several species, including the mouse, swine, ovine, bovine, and humans [3].

In our previous studies we evaluated LPA production and action in the bovine endometrium [9–11] and ovary [12, 13]. These studies demonstrated that LPA is a signaling molecule involved in luteal and endometrial functions that are relevant for pregnancy establishment in the cow. However, it is unknown whether LPA can be synthesized by early bovine embryos and whether LPA plays a role in bovine embryonic development, quality, and survival.

Although embryo development 4 is evolutionarily highly conserved, some critical steps of early development are species-specific. Human and mouse preimplantation embryo development shows significant differences in gene expression patterns, programs of epigenetic modification, susceptibility to genetic instability, and timing of embryo genome activation [14]. In relevant aspects, such as the timing of epigenetic reprogramming and embryonic genome activation, bovine embryos reflect more closely the scenario occurring in human embryos [15]. Additionally, bovine* in vitro* produced embryos can be generated from oocytes recovered from cow ovaries, collected* postmortem* at a local abattoir, thus eliminating ethical concerns regarding mice manipulation and euthanasia.

This study was designed to evaluate the main hypothesis that early bovine embryos are a source and a target of LPA signaling. To test this hypothesis, we evaluated embryonic transcription and expression of genes coding for enzymes of LPA synthesis pathways, and LPA receptors, and measured LPA concentrations in embryo culture medium. Additionally, we evaluated the effect of* in vitro* LPA stimulation on transcription of embryo quality marker genes and rate of blastocyst development.

## 2. Materials and Methods

### 2.1. In Vitro Embryo Production

Bovine embryos were produced* in vitro* as previously described [16]. Briefly, ovaries from Frisian crossbred heifers were collected* postmortem* at the local abattoir and transported to the laboratory at 37°C, within one hour. Cumulus-oocyte complexes (COCs) were obtained by aspiration of follicles with 2–8 mm diameter. COCs with at least three layers of compact cumulus cells and even cytoplasm were selected, washed, and placed in 400 *μ*L of maturation medium in 4-well dishes (25 COCs/well) overlaid with 400 *μ*L mineral oil and incubated at 39°C in a 5% CO_2_ in humidified air atmosphere for 24 hours. Following maturation, COCs were washed and placed in 4-well dishes containing 400 *μ*L of fertilization medium overlaid with 400 *μ*L mineral oil and coincubated with sperm at 39°C in a 5% CO_2_ in humidified air atmosphere for 48 hours. For* in vitro* insemination, frozen-thawed semen from one bull with previously proven* in vitro* and* in vivo* fertility was used throughout the experiment. After thawing, semen was recovered using the swim-up procedure. The sperm concentration per fertilization well was adjusted to 1×10^6^ sperm/mL and the day of* in vitro* insemination was considered as Day 0. On Day 2, cleavage stage embryos were denuded from remaining cumulus cells by vortexing and embryos with 4 or more blastomeres were selected for* in vitro* culture. These embryos were washed, placed in 4-well dishes (25 per well) containing 400 *μ*L of culture medium overlaid with 400 *μ*L mineral oil, and incubated in a 5% CO_2_ plus 5% O_2_ in humidified air atmosphere until Day 5 or Day 8. Embryos were evaluated for stage of development and morphological quality according to IETS guidelines [17]. Only embryos classified as quality grade 1 (excellent) or grade 2 (good) were selected for immunohistochemistry (IHC) and Real-Time PCR (qRT-PCR).

### 2.2. LPA Measurement in Embryo Culture Medium

Culture medium from Day 5 and Day 8 embryo cultures was recovered and stored at −80°C until LPA measurement, according to procedures described by Saulnier-Blache et al. [18]. Briefly, 1-Oleoyl-LPA contained in 400 *μ*L of culture medium was extracted with one volume of 1-butanol, and the reaction products were separated by two-dimensional thin-layer chromatography (2D-TLC) and autoradiographed. To convert radioactivity to picomoles, lipids visualized under iodine vapors were scraped and counted with 3 mL of scintillation cocktail. LPA concentrations of control culture medium (blank; mean concentrations = 95 pmol/mL, i.e., 38 pmol/well of 400 *μ*L) were subtracted from those of embryo culture medium to calculate final LPA concentrations (pmol/mL). This value was divided by the number of viable embryos present in wells at the end of culture. LPA concentrations were expressed as pmol/viable embryo.

### 2.3. LPA Stimulation of In Vitro Embryo Culture

At Day 2, embryos were randomly allocated to one of two experimental groups: (i) control, exposed to vehicle alone (PBS); and (ii) LPA, exposed to a LPA agonist (1 oleoyl-snglycero-3-lysophosphatidic acid sodium salt, Alexis) at a concentration of 10^−5^ M in PBS. Embryos were cultured until Day 8 and evaluated for stage of development and morphological quality. Only embryos classified as quality grade 1 (excellent) or grade 2 (good) were selected for RNA extraction.

### 2.4. RNA Extraction and Real-Time PCR Analysis

Embryonic RNA was extracted from five pools of 3 embryos using the Arcturus PicoPure RNA Isolation Kit (Applied Biosystems, Life Technologies) according to the manufacturer's instructions. DNA digestion was performed with the RNase-free DNase Set (Promega, Wood Hollow road, Madison, USA). Concentration and purity of RNA were determined spectrophotometrically at 260 nm and 280 nm (NanoDrop 1000, Thermo Scientific, USA), and samples were stored at −80°C until processing. Complementary DNA (cDNA) synthesis was obtained using the SuperScript III First-Strand Synthesis SuperMix (Invitrogen), the reverse transcriptase (RT) reaction being performed in a total reaction volume of 20 *μ*L. The obtained RT products were stored at −20°C until qRT-PCR amplification.

Target genes included genes coding for enzymes of the prostaglandins (PGs;* PTGS2*,* PGES*, and* PGFS*) and progesterone (P_4_;* 3*β*HSD*) synthesis pathways, genes associated with apoptosis (*BAX* and* BCL2*), and quality markers (*IGFR2* and* OCT4*), using* GAPDH* transcription as an internal control. This housekeeping gene was chosen using the NormFinder software, comparing three candidate genes:* GAPDH*,**β*-actin,* and* H2A.1* [19]. Primers were chosen using an online software package (http://bioinfo.ut.ee/primer3/) (Table 1) and were validated following the use of different concentrations and the observation that each pair generated a single amplicon with the predicted molecular size. The qRT-PCR was performed with an ABI Prism 7900 (Applied Biosystems, Life 12 Technologies, USA) sequence detection system using Maxima SYBR Green/ROX qPCR 13 Master Mix (number K0222, Fermentas, Thermo Scientific, USA). The PCR reactions were performed in 96-well plates. Each PCR reaction well (20 *μ*L) contained 2 *μ*L of RT product, 5 pmol/*μ*L of forward and reverse primers, and 10 *μ*L SYBR Green PCR master mix. The qRT-PCR was performed under the following conditions: 95°C for 10 min, followed by 40 cycles at 94°C for 15 sec and 60°C for 60 sec. Melting curves were acquired (15 s at 95°C, 30 s at 60°C, and 15 s at 95°C) to ensure that a single product was amplified in the reaction. In order to exclude the possibility of genomic DNA contamination in RNA samples, the reactions were also run either on blank-only buffer samples or in absence of the reverse transcriptase enzyme. The specificity of the PCR products for all examined genes was confirmed by gel electrophoresis and by sequencing. Data regarding relative mRNA quantification was analyzed with the Real-Time PCR Miner algorithm [20].

### 2.5. Immunofluorescence

Selected embryos were fixed in 4% paraformaldehyde for 1 h at 4°C, washed in PBS, and permeabilized with 0.5% Triton X-100 for 10 min at room temperature. After washing in PBS, embryos were incubated for 1 h in blocking solution (Novocastra Protein Block, ref^a^ RE7102, Novocastra Laboratories Ltd., Newcastle upon Tyne, UK). This blocking step was followed by overnight incubation at 4°C with the following antibodies (Santa Cruz Biotechnology), diluted 1 : 100 in blocking solution: rabbit polyclonal anti-ATX (sc-66813), PLA2 (sc438), LPAR1 (sc-22207), LPAR2 (sc-25490), LPAR3 (sc25492), and LPAR4 (sc-46021). Antibodies' specificity in bovine tissue was previously confirmed, through WB and IHC [12, 21]. Negative control sections were incubated with normal rabbit irrelevant IgG (concentration 1 : 100, sc-2027). After washing in PBS, embryos were incubated for 1 h at room temperature with Alexa Fluor 488 donkey anti-rabbit IgG antibody (concentration 1 : 300 in blocking solution, A-21206, Invitrogen, Life Technologies, Foster City, CA, USA). Following a final washing in PBS, embryos were mounted in Vectashield with DAPI (ref. H-1200, Vector Laboratories, Brunschwig Chemie, Amsterdam, Netherlands) and examined under a Leica DMRA fluorescence microscope.

### 2.6. Statistical Analysis

Data were analyzed through the statistical software GraphPad PRISM 5.0 (GraphPad Software, Inc., La Jolla, CA, USA). Data are presented as mean ± SEM. Quantitative data were analyzed by Student's* t*-test for independent pairs, whereas categorical data were analyzed by Fisher's exact test. The differences were considered statistically significant at the 95% confidence level (*P* < 0.05).

## 3. Results

### 3.1. Late Cleavage and Blastocyst Stage Bovine Embryos Transcribed and Expressed Genes Coding for Enzymes of the LPA Synthesis Pathway and Produced LPA into Culture Medium

**Figure 1:**
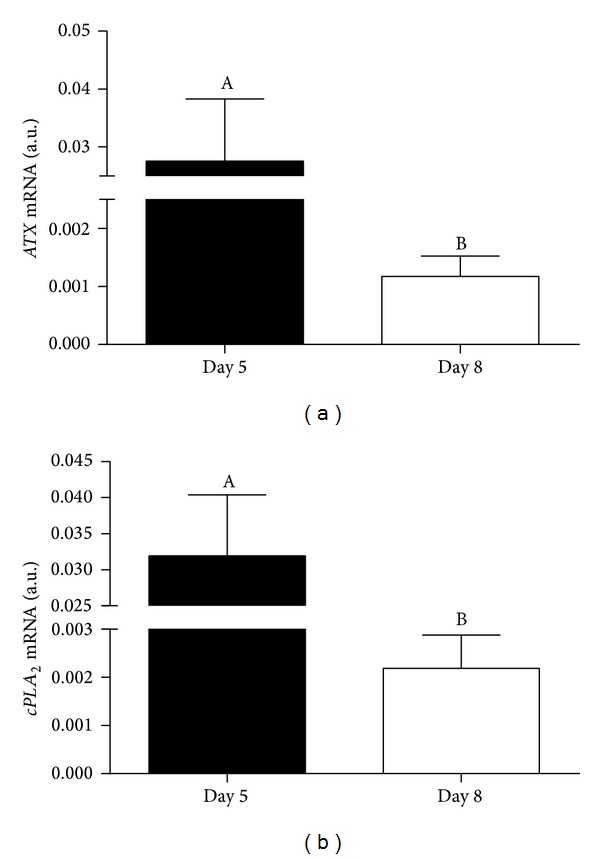
Transcription levels of genes coding for LPA synthesis enzymes ((a)* ATX*; (b)* cPLA*_*2*_) in late cleavage (Day 5) and blastocyst (Day 8) stage bovine* in vitro* produced embryos. Columns with different superscripts differ significantly: AB, *P* < 0.05.

**Figure 2:**
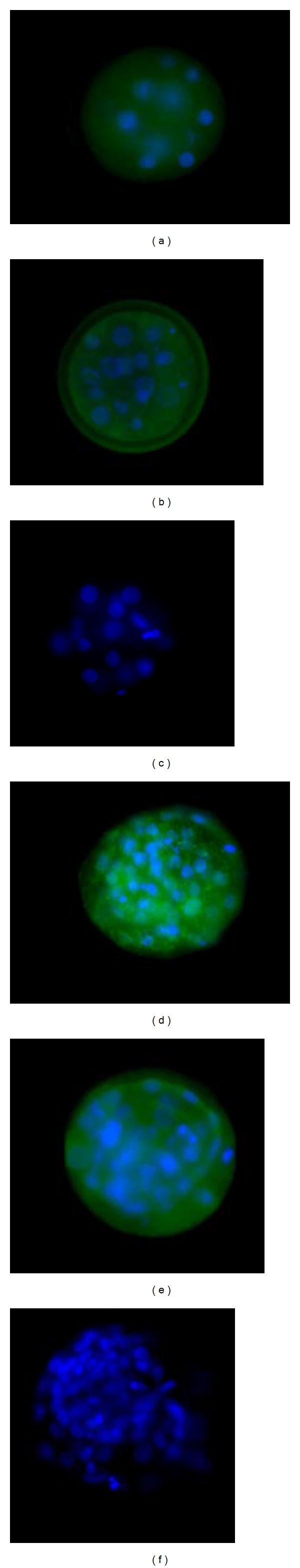
Expression of LPA synthesis enzymes ATX ((a) and (d)) and cPLA_2_ ((b) and (e)) in late cleavage (Day 5; upper row) and blastocyst (Day 8; bottom row) stage bovine* in vitro* produced embryos. (c) and (f) negative controls; 200x magnification.

**Figure 3:**
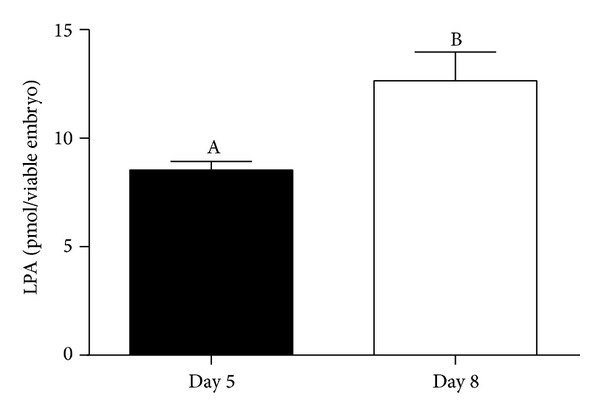
LPA production by late cleavage (Day 5) and blastocyst (Day 8) stage bovine* in vitro *produced embryos. LPA concentrations represent the accumulated production since Day 2 and are expressed as pmol/viable embryo. Columns with different superscripts differ significantly: AB, *P* < 0.05.

As shown in Figure 1, transcription of genes coding for enzymes of the LPA synthesis pathway ((a)* ATX* and (b)* cPLA*_*2*_) was detected in Day 5 and Day 8 embryos. Transcription levels of both genes were significantly higher in late cleavage than in blastocyst stage embryos. Expression of ATX and cPLA_2_ proteins was observed in cells of late cleavage and blastocyst stage embryos (Figure 2). LPA was detected in culture medium of Day 5 and Day 8 embryos. However, LPA concentrations were significantly higher in medium recovered from blastocyst culture than in medium recovered from late cleavage embryos' culture (Figure 3).

### 3.2. Late Cleavage and Blastocyst Stage Embryos Transcribed and Expressed Genes Coding for LPA Receptors (LPAR1–4)

**Figure 4:**
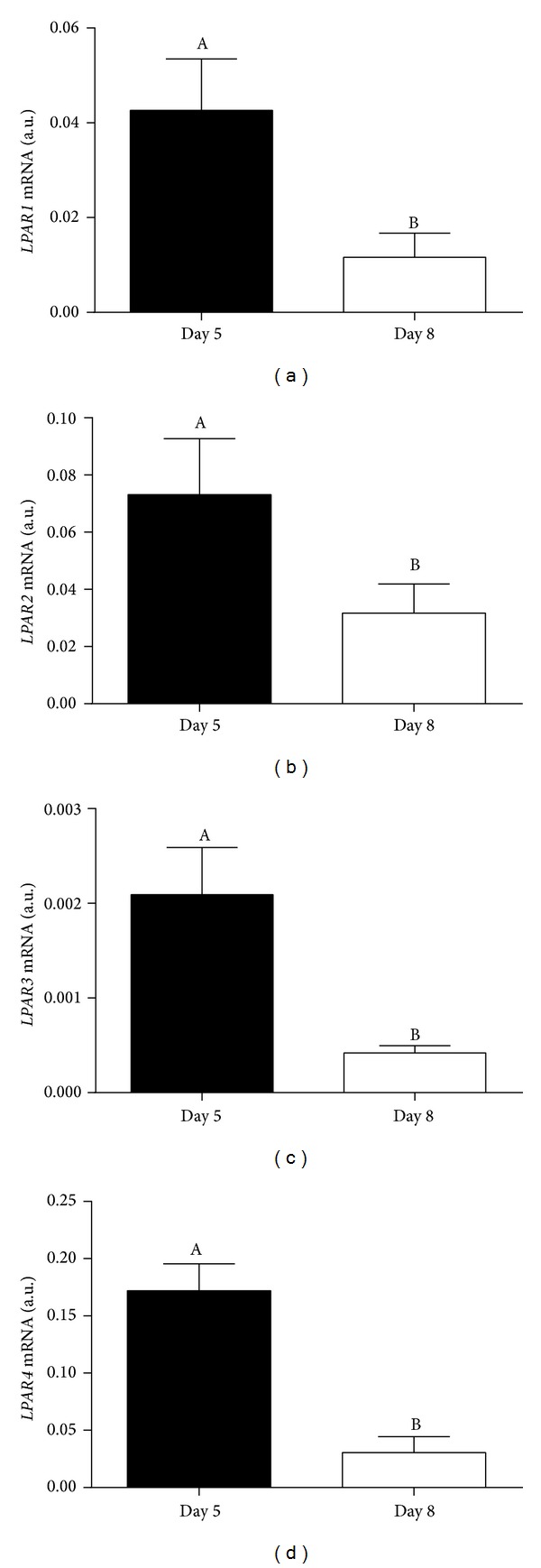
Transcription levels of genes coding for LPA receptors ((a)* LPAR1*; (b)* LPAR2*; (c)* LPAR3*; (d)* LPAR4*) in late cleavage (Day 5) and blastocyst (Day 8) stage bovine* in vitro* produced embryos. Columns with different superscripts differ significantly: AB, *P* < 0.05.

**Figure 5:**
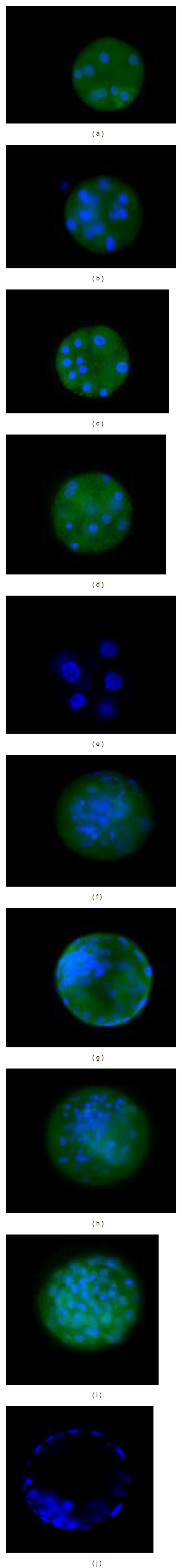
Expression of LPARs (LPAR1, LPAR2, LPAR3, and LPAR4) in late cleavage (Day 5; (a), (b), (c), and (d), resp.) and blastocyst (Day 8; (f), (g), (h), and (i), resp.) stage bovine* in vitro* produced embryos. (e) and (j) negative controls; 200x magnification.

As shown in Figure 4, transcription of genes coding for LPAR1 (a), LPAR2 (b), LPAR3 (c), and LPAR4 (d) was detected in Day 5 and Day 8 bovine embryos. Late cleavage stage embryos showed significantly higher mRNA abundance of all LPARs than blastocyst stage embryos. Expression of LPAR1–4 proteins was observed in Day 5 and Day 8 embryos (Figure 5).

### 3.3. LPA Supplementation of Culture Medium Had No Effect on Embryo Development and Morphological Quality

As shown in Table 2, the blastocyst rates on Day 8 were similar in LPA-stimulated and control embryos. The proportions of quality grade 1 and grade 2 Day 8 blastocysts were also similar in the LPA and control groups.

### 3.4. LPA Supplementation of Culture Medium Had No Effect on Blastocyst Transcription Levels of Genes Coding for Enzymes of Prostaglandin and Steroidogenic Synthesis Pathways

**Figure 6:**
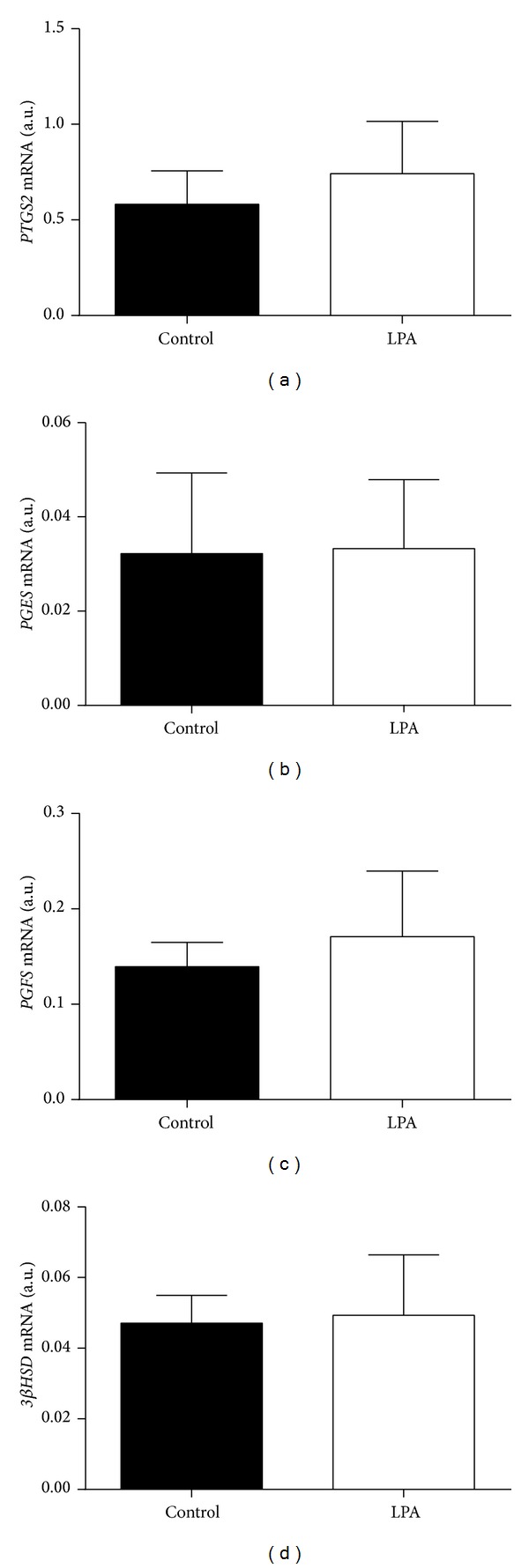
Effect of LPA stimulation on transcription levels of genes coding for PG ((a)* PTGS2*; (b)* PGES*; (c)* PGFS*) and P_4_ ((d)* 3*β*-HSD*) synthesis enzymes in bovine* in vitro* produced blastocysts.

As shown in Figure 6, transcription levels of* PTGS2*,* PGES*,* PGFS,* and* 3*β*HSD* were similar in Day 8 LPA-stimulated and control embryos.

### 3.5. LPA Supplementation of Culture Medium Affected Blastocyst Transcription Levels of Quality Marker Genes

**Figure 7:**
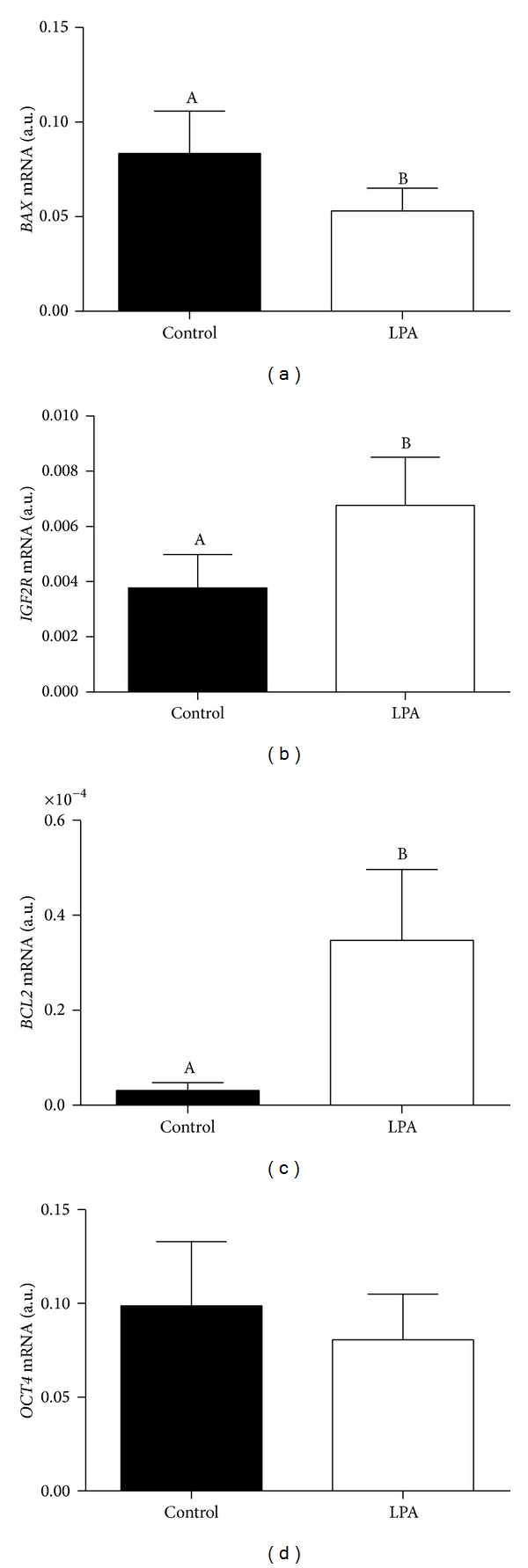
Effect of LPA stimulation on transcription levels of genes coding for embryo quality markers ((a)* BAX*; (b)* IGF2R*; (c)* BCL2*; and (d)* OCT4*) in bovine* in vitro* produced blastocysts. Columns with different superscripts differ significantly: AB, *P* < 0.05.

As shown in Figure 7, LPA-stimulated blastocysts showed a significantly lower transcription level of* BAX* and significantly higher transcription levels of* BCL2* and* IGF2R* than control blastocysts. Transcription levels of* OCT4* were similar in LPA-stimulated and control blastocysts.

## 4. Discussion

This study demonstrates for the first time that late cleavage and blastocyst stage bovine embryos are able to transcribe and express genes coding for enzymes of LPA synthesis pathways (*ATX* and* cPLA*_*2*_) and produce LPA into culture medium. This evidences that the early embryo is able to use both pathways of LPA production. Interestingly, the PLA_2_ pathway is mainly observed during inflammatory events [4, 5], which may lead to the speculation that early embryo-maternal signaling includes an inflammatory component. Transcript abundance was higher in late cleavage (Day 5) than in blastocyst (Day 8) stage embryos.* In vivo*, Day 5 cleaving uncompacted morulae travel through the oviduct, whereas Day 8 expanding/ecloding blastocysts are shed in the endometrium. In mice, LPA stimulated oviduct smooth muscle contraction and ovum transport through the oviduct [22]. In bovine, active LPA production by embryos of later stages of cleavage may also indicate a paracrine role in the oviduct, to enhance embryo transport into the uterus.

Embryonic LPA production was higher in blastocyst than in late cleavage stage embryos. This may reflect the higher cell content of blastocysts compared to late cleavage stages and/or the accumulated production from Day 5 to Day 8. Active LPA production by bovine blastocysts is suggestive of an early paracrine interaction between the embryo and the uterus. We demonstrated that the bovine uterus is a source and a target of LPA, through LPAR1-mediated actions [9, 10].* In vitro* LPA stimulation of endometrial cells recovered from cows either on Days 8–10 of the estrous cycle or on Days 8–10 of pregnancy showed that LPA inhibited endometrial PGF2*α* production only in early pregnancy [23]. Taken together, these results lead us to the proposal that blastocyst LPA paracrine signaling in the uterus attenuates endometrial PGF2*α* production, contributing to luteal maintenance and maternal recognition of pregnancy.

Late cleavage and blastocyst stage bovine embryos transcribed and expressed genes coding for LPARs (1–4), and gene transcription was higher in late cleavage than in blastocyst stage embryos. This indicates that preeclosion embryos are also a target for LPA autocrine (within the embryo) and paracrine (from the oviduct/uterus) actions. In mice, embryonic transcription of* LPAR1-2* was detected in differentiating blastocysts [24] and* LPAR1–4* transcripts were detected in 6.5 to 10.5 dpc embryos [25]. In sheep, transcription and expression of LPARs were evaluated in Day 12 to Day 18 conceptuses [26].* LPAR1* and* LPAR3* transcripts peaked on Day 14, whereas expression of LPAR proteins peaked on Day 17.

Uterine concentrations of LPA were estimated to be 0.8–1 *μ*M in the ovine [26] and porcine [27].* In vitro*, LPA synthesis is decreased due to the lower availability of substrate (lysophosphatidylcholine), and synthesized LPA is degraded along culture time [26]. This also holds true to exogenously added LPA; therefore we chose a high LPA treatment concentration to counterbalance degradation along culture time. In this study, LPA stimulation had no effect on embryonic development and morphological quality. In mice, LPA stimulation at the pronuclear stage improved blastocyst rate, but stimulation at later stages had no effect on development [28]. This is in line with the present results. Therefore, the embryonic rate of development is probably not regulated by the paracrine action of oviduct/uterus secreted LPA.

Blastocyst stage bovine embryos are able to transcribe genes coding for PGs synthesis pathway enzymes and for P_4_ synthesis enzymes and produce PGs (PGF_2*α*_ and PGE_2_) and P_4_ into culture medium [16].* In vitro*, we found a stimulatory effect of LPA on P_4_ synthesis and interferon (IFN)*τ* action in bovine luteal cells [12] and on luteotropic PGE_2_ secretion in the bovine endometrium [9, 10].* In vivo*, we also found that LPA infusion prevented spontaneous luteolysis, prolonged the functional lifespan of the corpus luteum, and stimulated luteotropic PGE_2_ synthesis in heifers [11]. Additionally, administration of a LPARs antagonist decreased pregnancy rate in cows [11]. These results indicate that LPA is relevant in early pregnancy maintenance in the bovine, mainly through its luteotropic action. In the ovine, LPA stimulation of trophectoderm cells in culture induced a 2-fold increase in PGF_2*α*_ and PGE_2_ synthesis, not associated with an increase in* cPLA2*α** and* PTGS2* transcription [26]. Authors [26] suggested that LPA-mediated signaling occurs through the release of PGs, probably due to a stimulation of arachidonic acid mobilization. Here, LPA stimulation had no effect on transcription of genes coding for enzymes of PG synthesis (*PTGS2*,* PGES*, and* PGFS*) and steroidogenic (*3*β*HSD*) pathways. However, we cannot exclude the possibility of an LPA-induced embryonic release of PGs independent of* PTGS2*,* PGES,* and* PGFS* mRNA abundance, as we did not measure PGs in culture medium. Also, the functional role of embryonic P_4_ production remains unclear. Here, LPA stimulation had no effect on* 3*β*HSD* transcription, contrary to what was observed in luteal cells [12].

LPA stimulation induced a decrease in blastocyst transcription of* BAX* and an increase in transcription of* BCL2*. Balanced cellular expression of BAX and BCL2, pro- and antiapoptotic factors, respectively, is determinant for the control of cell survival [29]. The BAX/BCL2 protein ratio was decreased in fragmented bovine blastocysts, leading to the suggestion that this ratio might be used to evaluate the tendency of oocytes and embryos towards either survival or apoptosis [30]. Also, LPA stimulation of cell culture regulated expression of BCL2 and BAX, enhancing cell survival [31–33]. In bovine cultured luteal cells, LPA stimulation inhibited TNF*α* and IFN*γ* (proapoptotic mediators) induced apoptosis; this effect was obtained through the inhibition of BAX, TNFR1, Fas, FasL, and caspase 3 activity [34]. Here, blastocyst gene transcription was shifted towards high BCL2 and low BAX levels by LPA stimulation. Although LPA treatment did not affect embryo development and quality until Day 8, inhibition of apoptosis in blastocyst cells may be beneficial for subsequent* in vivo* survival.

We evaluated LPA stimulation effect on blastocyst transcription of two embryo quality marker genes,* IGF2R* and* OCT4*. Several studies reported expression of IGF system genes (*IGF1*,* IGF2*,* IGF1R*, and* IGF2R*) in bovine embryos [35–39]. IGF-system genes were proposed as bovine embryo quality marker genes, as transcription of these genes was associated with morphological assessment and growth potential of embryos [35]. In the present study, LPA stimulated blastocyst* IGF2R* transcription. Again, although LPA stimulation did not affect embryo development and quality until Day 8, stimulation of blastocyst growth may be beneficial for subsequent* in vivo* survival.

In mice, OCT4-deficient embryos develop to blastocyst, but ICM cells lose pluripotency [40]. Although transcription factor OCT4 is a cornerstone of pluripotency, a recent study [41] showed that OCT4 major activity in mouse blastocysts is to support primitive endoderm differentiation. In bovine, IVF and SCNT blastocysts showed significantly different expression of OCT4 [42]. Here, LPA stimulation had no effect on* OCT4* blastocyst transcription, which indicates that LPA does not interfere with OCT4 pathway mediated blastocyst differentiation.

In conclusion, late cleavage and blastocyst stage bovine embryos transcribed and expressed genes coding for LPA synthesis enzymes and produced LPA into culture medium. Additionally, late cleavage and blastocyst stage bovine embryos transcribed and expressed genes coding for LPARs. This turns early bovine embryos into a potential source and target of autocrine/paracrine LPA mediated cell signaling. LPA stimulation had no effect on* in vitro* embryo development and quality until Day 8 but affected blastocyst transcription of apoptosis (*BAX*,* BCL2*) and growth (*IGF2R*) related genes. Inhibition of apoptosis and promotion of growth in blastocyst cells may be relevant for subsequent* in vivo* embryo survival. Altogether, the present results lead us to propose that LPA mediated cell signaling may operate in an auto-, paracrine way during bovine early embryonic development, being involved in early embryo-maternal interactions leading to embryo survival. Due to similarities between bovine and human early embryonic development [15], results described here may also reflect events occurring in the human embryo.

## Figures and Tables

**Table 1:** Target genes and primer pair sequences used in qRT-PCR.

Gene	Primer sequence (5′-3′)	Fragment size, bp	GenBank accession number
*LPAR1*	ACGGAATCGGGATACCATGA CCAGTCCAGGAGTCCAGCAG	86	NM_174047.2
*LPAR2*	TTCTATGTGAGGCGGCGAGT AGACCATCCAGGAGCAGCAC	161	NM_001192235.1
*LPAR3*	TCCAACCTCATGGCCTTCTT GACCCACTCGTATGCGGAGA	101	NM_001192741.2
*LPAR4*	CCACAGTACCTCCAGAAAGTTCA TTGGAATTGGAAGTCAATGAATC	192	NM_001098105.1
*ATX*	ACCCCCTGATTGTCGATGTG TCTCCGCATCTGTCCTTGGT	120	NM_001080293.1
*PLA2*	CTGCGTGCCACAAAAGTGAC TCGGGGGTTGAAGAGATGAA	92	NM_001075864.1
*BCL2*	GAGTTCGGAGGGGTCATGTG GCCTTCAGAGACAGCCAGGA	203	NM_001166486.1
*BAX*	GTGCCCGAGTTGATCAGGAC CCATGTGGGTGTCCCAAAGT	126	NM_173894.1
*3*β*HSD*	TCCCGGATGAGCCTTCCTAT ACTAGGTGGCGGTTGAAGCA	116	NM_174343.2
*COX-2*	TGGGTGTGAAAGGGAGGAAA AAGTGCTGGGCAAAGAATGC	127	AF004944.1
*PTGES*	CCGAGGACGCTCAGAGACAT AAAGCCCAGGAACAGGAAGG	122	NM_174443.2
*PGFS*	GGAGGACCCCAGGATCAAAG CTCAGCAATGCGTTCAGGTG	130	S54973.1
*IGF2R*	ACCTCCGATCCTCAATCCCA TGTAGTTGAAGTGCCGGTCC	89	NM_174352.2
*OCT4*	GAGAAAGACGTGGTCCGAGTG GACCCAGCAGCCTCAAAATC	101	NM_174580.2
*GAPDH*	CACCCTCAAGATTGTCAGCA GGTCATAAGTCCCTCCACGA	103	NM_001034034.2

**Table 2:** Effect of LPA supplementation on bovine *in vitro* embryo development and quality.

Group	*n*	Day 8 embryos *n* (%)	Qualities 1 and 2 *n* (%)
Control	304	101 (33)	92 (91)
LPA	329	110 (33)	103 (93)
